# Lower body strength and body composition in female football

**DOI:** 10.1038/s41598-025-94041-x

**Published:** 2025-03-17

**Authors:** Cíntia França, Carolina Saldanha, Francisco Martins, Marcelo de Maio Nascimento, Adilson Marques, Andreas Ihle, Hugo Sarmento, Pedro Campos, Élvio Rúbio Gouveia

**Affiliations:** 1 Department of Physical Education and Sport, University of Madeira, 9020-105 Funchal, Portugal; 2 LARSYS, Interactive Technologies Institute, 9020-105 Funchal, Portugal; 3 Faculty of Sport Sciences and Physical Education, CIDAF, University of Coimbra, 3040-248 Coimbra, Portugal; 4 Department of Physical Education, Federal University of Vale do São Francisco, Petrolina, 56304-917 Brazil; 5 Swiss Center of Expertise in Life Course Research LIVES, 1227 Carouge, Switzerland; 6 Faculty of Human Kinetics, CIPER, University of Lisbon, Cruz Quebrada, 1499-002 Lisboa, Portugal; 7 Environmental Health Institute (ISAMB), Faculty of Medicine, University of Lisbon, 1649-020 Lisbon, Portugal; 8 Department of Psychology, University of Geneva, 1227 Carouge, Switzerland; 9 Center for the Interdisciplinary Study of Gerontology and Vulnerability, University of Geneva, 1227 Carouge, Switzerland; 10 WoWSystems Informática Lda, 9050-100 Funchal, Portugal

**Keywords:** Soccer, Explosive strength, Isokinetic strength, Peak torque, Intra-limb asymmetry, Inter-limb asymmetry, Physiology, Health occupations

## Abstract

Lower-body strength plays a crucial role in football performance and injury prevention, and thus, monitoring of strength variables has become crucial in the training process. This study aims to (1) assess knee muscle strength performance through intra- and inter-limb asymmetries and (2) examine the relationships between knee muscle strength, body composition, and vertical jump performance (squat jump and countermovement jump). Twenty-seven semiprofessional female football players (21.5 ± 4.9 years) were evaluated for body composition, isokinetic knee muscle strength (60º/s and 180º/s), and vertical jump tasks. Peak torque (PT), peak torque/body weight (PT/BW), bilateral strength deficit, and the hamstring-to-quadriceps strength ratio (H/Q) for knee extensors (KE) and flexors (KF) in both the preferred and non-preferred legs. The H/Q ratio and the bilateral strength deficit revealed no significant intra- or inter-limb asymmetries in knee muscle strength. Strong correlations were found between vertical jump performance and KE strength at both 60º/s (*p* ≤ 0.01) and 180º/s (*p* ≤ 0.01). Additionally, a significant negative correlation was observed between vertical jump performance and body fat percentage (*p* ≤ 0.01). These findings highlight the critical role of knee muscle strength in explosive tasks and underline the negative impact of higher body fat on lower-body strength performance.

## Introduction

Football is characterized by its high-intensity efforts, such as jumps, change of direction, and sprint actions^1^. According to the literature, high-intensity activities are strongly related to lower-body strength levels^2^. This heightened intensity may increase the players’ risk of injury, resulting in absence from future training sessions or competitions^3^. Previous research on injury occurrence among male football players has reported the quadriceps as the most affected body zone by injury, followed by the hamstring and adductors^4,5^. In female football, evidence suggests that the knee is the body part most frequently affected by injury (ranging from 16 to 32% of all time-loss injuries), followed by ankle sprains^6^. Considering the influence of lower-body strength on game performance and injury prevention^2^, monitoring players’ strength variables has become an important part of the training process.

Based on the injury risk in the knee joint, the isokinetic dynamometer has been a valuable instrument for coaches and their staff in players’ assessment and monitoring. This device has been considered the gold standard for accurately measuring peak torque (PT) values of knee flexors (KF) and knee extensors (KE) throughout both the concentric and eccentric phases. Its efficacy has led to suggestions for its use as a monitoring tool to track the incidence of non-contact injuries, such as muscle strains and non-contact anterior cruciate ligament ruptures (Croisier et al., 2008; Weiss & Whatman, 2015). Moreover, the hamstring-to-quadriceps ratio (H/Q ratio) has been used to evaluate intra-limb asymmetry^7^. Although its predictive accuracy has recently been questioned, a low hamstring-to-quadriceps ratio (H/Q ratio) during concentric action has been related to an increased risk of acute lower-body injuries^8^.

In the literature, several reports of muscle strength imbalances between the preferred leg (PL) and the non-preferred leg (NPL) performance are available among football players^9,10^. Inter-limb differences of at least 15% were associated with injury occurrence among 138 female collegiate athletes^9^. Although it has been acknowledged that injury occurrence is multifactorial^11^, which limits the interpretation of both H/Q ratio scores and the percentage of limb muscle strength differences, monitoring intra and inter-limb asymmetries remains essential during players’ development^12^, and as a potential marker for injury^13^.

Meantime, the isokinetic strength assessment has also been described as an indicator of lower-body explosive strength based on the application of vertical jumps^14,15^. Higher correlation coefficients were observed from the slow to the faster isokinetic testing velocities and the squat jump performance in male athletes^14,16^. Isokinetic PT can predict jumping work, accounting for 75% and 69% of the variance observed in the countermovement jump (CMJ) and squat jump (SJ) performance^15^. Additionally, several reports are available in the literature on the positive relationship between isokinetic strength and vertical performance, particularly the CMJ^16–18^. However, among female footballers, investigations on the relationship between isokinetic strength and vertical jumps are still scarce, with only one study being found on this topic, reporting moderate (-0.06 > *r* < 0.24) relationships between PT, CMJ, and SJ^19^.

In the meantime, the body composition analysis focuses on fat percentage (BF%) by its negative influence on sports performance, particularly in tasks that demand rapid body displacement (i.e., sprints, jumps, change of directions)^20,21^. Research in youth male football showed significant and positive correlations between BF% and sprint ability (10- and 35-m sprint), reinforcing that higher BF% is associated with higher sprint time^20^. In female football, a recent study on national-level Finnish footballers describes significant and negative relationships between BF% and match running performance (total distance, high-intensity running, and very high-intensity running)^22^. Overall, the evidence related to the detrimental effect of BF% on physical performance is well-established in male football. However, details on this topic are still needed among the female population to better understand the relationship between these variables, which could support better training and intervention strategies.

Considering the influence of muscle strength on game performance, assessing KF and KE strength levels could be fundamentally important for coaches and their staff to develop effective training programs. Although some evidence is available on football players’ knee muscle strength and vertical jump performance, most of the research has privileged males. Indeed, details concerning lower-body strength characteristics and their relationship with body composition among female players are still lacking in the literature. Research on this topic is crucial to provide insights that might be useful for training planning and periodization on a growing sport worldwide. Thus, this study addresses two main purposes: (1) to evaluate knee muscle strength performance according to intra- and inter-limb asymmetries and (2) to examine the relationships between knee muscle strength, body composition, and vertical jump performance among female football players.

## Results

**Fig. 1:**
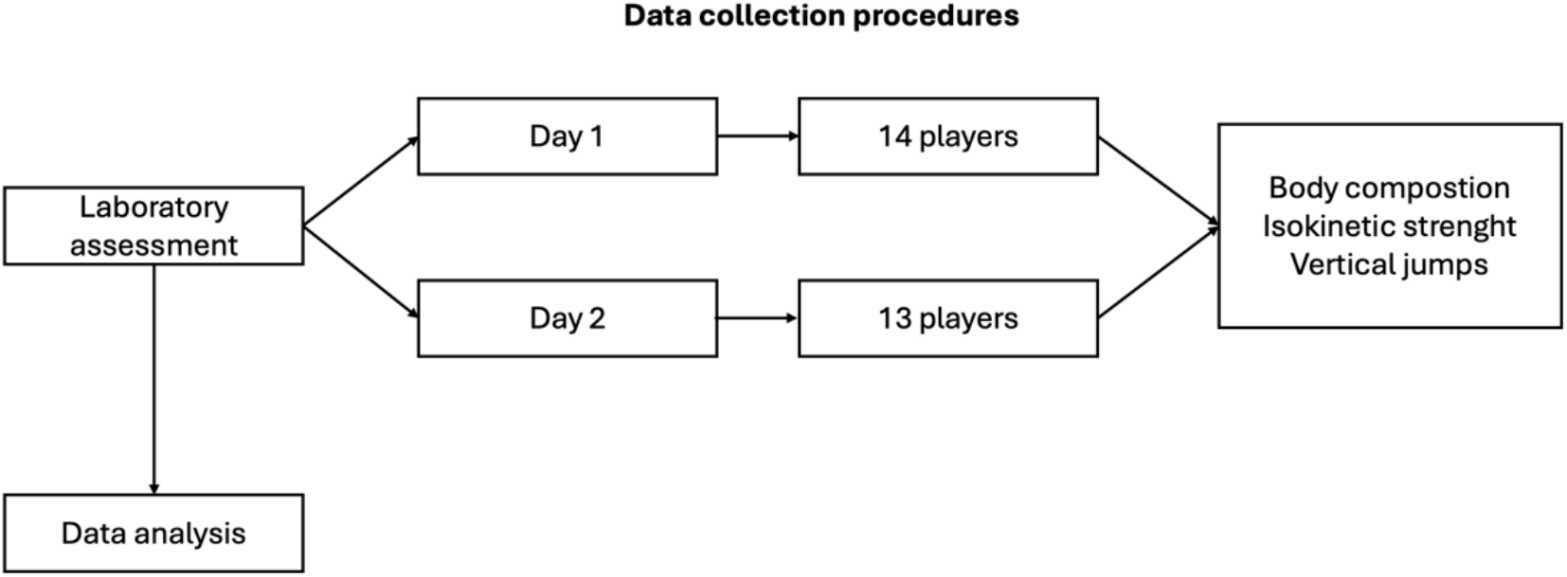
Flowchart illustrating data collection procedures.

Table 1 resumes descriptive statistics for participants’ age, body composition, and vertical jump performance (Fig. 1).

**Table 1:** Descriptive statistics for age, anthropometry and body composition, speed, and lower-body explosive strength tests.

Variables	Mean ± Standard deviation
Age (years)	21.5 ± 4.9
Stature (cm)	165.3 ± 5.7
Bodyweight (kg)	61.3 ± 7.8
Body fat (%)	21.6 ± 5.1
Fat-free mass (kg)	47.9 ± 5.0
Countermovement jump (cm)	26.9 ± 3.6
Squat jump (cm)	26.1 ± 3.7

Table 2 presents the descriptive statistics and comparison results for inter-limb performance in knee muscle strength. Overall, the scores attained with the PL are superior to the NPL in knee muscle performance. However, the differences were not statistically significant. The same pattern was verified for the H/Q ratio, with no substantial differences being found at 60º/s and 180º/s angular velocities.

**Table 2:** Descriptive statistics for knee muscle strength performance and comparison results between the PL and the NPL.

Variables	PL	NPL	t	*p*
60º/s angular velocity				
KF PT (N·m)	85.7 ± 12.2	83.1 ± 14.2	1.250	0.223
KF PT/BW (N·m)	1.40 ± 0.18	1.36 ± 0.23	1.197	0.242
KE PT (N·m)	153.4 ± 17.6	153.9 ± 18.5	-0.247	0.807
KE PT/BW (N·m)	2.52 ± 0.37	2.53 ± 0.37	-0.219	0.828
H/Q ratio (%)	54.2 ± 13.1	52.1 ± 12.5	1.719	0.098
180º/s angular velocity				
KF PT (N·m)	65.0 ± 12.4	62.9 ± 12.4	1.185	0.247
KF PT/bodyweight (N·m)	1.02 ± 0.29	0.99 ± 0.28	1.082	0.289
KE PT (N·m)	103.3 ± 17.1	102.0 ± 15.6	0.742	0.465
KE PT/bodyweight (N·m)	1.64 ± 0.46	1.62 ± 0.44	0.668	0.510
H/Q ratio (%)	61.2 ± 15.8	59.7 ± 15.3	0.929	0.362

*PL* preferred leg,* NPL* non-preferred leg,* KF* knee flexors,* KE* knee extensors,* PT* peak torque,* PT/BW* peak torque normalized by bodyweight,* H/Q* hamstrings-to-quadriceps ratio.

Table 3 summarizes the results of Pearson product-moment correlation coefficients to investigate the relationship between vertical jump performance and knee muscle strength. As expected, knee muscle strength variables and vertical jump testing showed a significant positive relationship. For the CMJ, the strongest correlations were observed with KE variables, both at 60º/s (PL: *r* = 0.58, *p* ≤ 0.01; NPL: *r* = 0.59, *p* ≤ 0.01) and 180º/s (PL: *r* = 0.63, *p* ≤ 0.01; NPL: *r* = 0.60, *p* ≤ 0.01). The same pattern was seen for the SJ performance at 60º/s (PL: *r* = 0.65, *p* ≤ 0.01; NPL: *r* = 0.66, *p* ≤ 0.01) and 180º/s (PL: *r* = 0.65, *p* ≤ 0.01; NPL: *r* = 0.63, *p* ≤ 0.01).

**Table 3:** Correlation coefficients between vertical jump tasks and knee muscle strength.

Variables	1.	2.	3.	4.	5.	6.	7.	8.	9.	10.
1. CMJ	–	0.95**	0.44*	0.42*	0.58**	0.59**	0.52**	0.46*	0.63**	0.60**
2. SJ		–	0.53**	0.49**	0.65**	0.66*	0.56**	0.51**	0.65**	0.63**
3. KF PT/BW (PL 60º/s)			–	0.88**	0.82**	0.82**	0.85**	0.86**	0.76**	0.79**
4. KF PT/ BW (NPL 60º/s)				–	0.78**	0.85**	0.78**	0.91**	0.76**	0.84**
5. KE PT/ BW (PL 60º/s)					–	0.95**	0.80**	0.78**	0.93**	0.90**
6. KE PT/ BW (NPL 60º/s)						–	0.74**	0.77**	0.91**	0.93**
7. KF PT/ BW (PL 180º/s)							–	0.85**	0.82**	0.79**
8. KF PT/ BW (NPL 180º/s)								–	0.78**	0.84**
9. KE PT/ BW (PL 180º/s)									–	0.94**
10. KE PT/ BW (NPL 180º/s)										–

*CMJ* countermovement jump,* SJ* squat jump,* KF* knee flexors,* KE* knee extensors,* PT/BW* peak torque normalized by bodyweight,* PL* preferred leg,* NPL* non-preferred leg.

* *p* ≤ 0.05, ** *p* ≤ 0.01.

Tables 4 and 5 present the results of Pearson product-moment correlation coefficients regarding body composition, knee muscle strength, and vertical jump performance. No significant statistical relationships were seen between knee muscle strength variables and body composition parameters (*p* ≥ 0.05). However, bodyweight and BF% appeared to be negative influencers of PT outputs.

On the other hand, significant and negative relationships were found between BF% and jump tasks (CMJ: *r* = −0.47, *p* ≤ 0.01; SJ: *r* = −0.42, *p* ≤ 0.01), suggesting that higher BF% results in decreased jump height.

**Table 4:** Correlation coefficients between knee muscle strength and body composition variables.

Variables	1.	2.	3.	4.	5.	6.	7.	8.	9.	10.	11.
1. KF PT/BW (PL 60º/s)	–	–	–	–	–	–	–	–	−0.04	0.05	−0.09
2. KF PT/ BW (NPL 60º/s)	–	–	–	–	–	–	–	–	−0.11	0.03	−0.19
3. KE PT/ BW (PL 60º/s)	–	–	–	–	–	–	–	–	−0.20	-0.33	−0.31
4. KE PT/ BW (NPL 60º/s)	–	–	–	–	–	–	–	–	−0.19	-0.04	-0.28
5. KF PT/ BW (PL 180º/s)	–	–	–	–	–	–	–	–	−0.11	0.03	-0.19
6. KF PT/ BW (NPL 180º/s)	–	–	–	–	–	–	–	–	−0.11	0.06	−0.23
7. KE PT/ BW (PL 180º/s)	–	–	–	–	–	–	–	–	−0.23	−0.02	−0.37
8. KE PT/ BW (NPL 180º/s)	–	–	–	–	–	–	–	–	−0.26	−0.06	−0.37
9. Bodyweight	–	–	–	–	–	–	–	–	–	–	–
10. FFM	–	–	–	–	–	–	–	–	–	–	–
11. BF%	–	–	–	–	–	–	–	–	–	-	–

*KF* knee flexors,* KE* knee extensors,* PT/BW* peak torque normalized by bodyweight,* PL* preferred leg,* NPL* non-preferred leg,* FFM* fat-free mass,* BF%* body fat percentage.

**Table 5:** Correlation coefficients between vertical jump tasks and body composition variables.

Variables	1.	2.	3.	4.	5.
1. CMJ	–	0.95**	−0.25	0.02	−0.47**
2. SJ		–	0.17	0.08	−0.42**
3. Bodyweight			−	0.83**	0.52**
4. FFM				−	−0.03
5. BF%					–

*CMJ* countermovement jump,* SJ* squat jump,* FFM* fat-free mass,* BF%* body fat percentage.

** *p* ≤ 0.01.

**Fig. 2:**
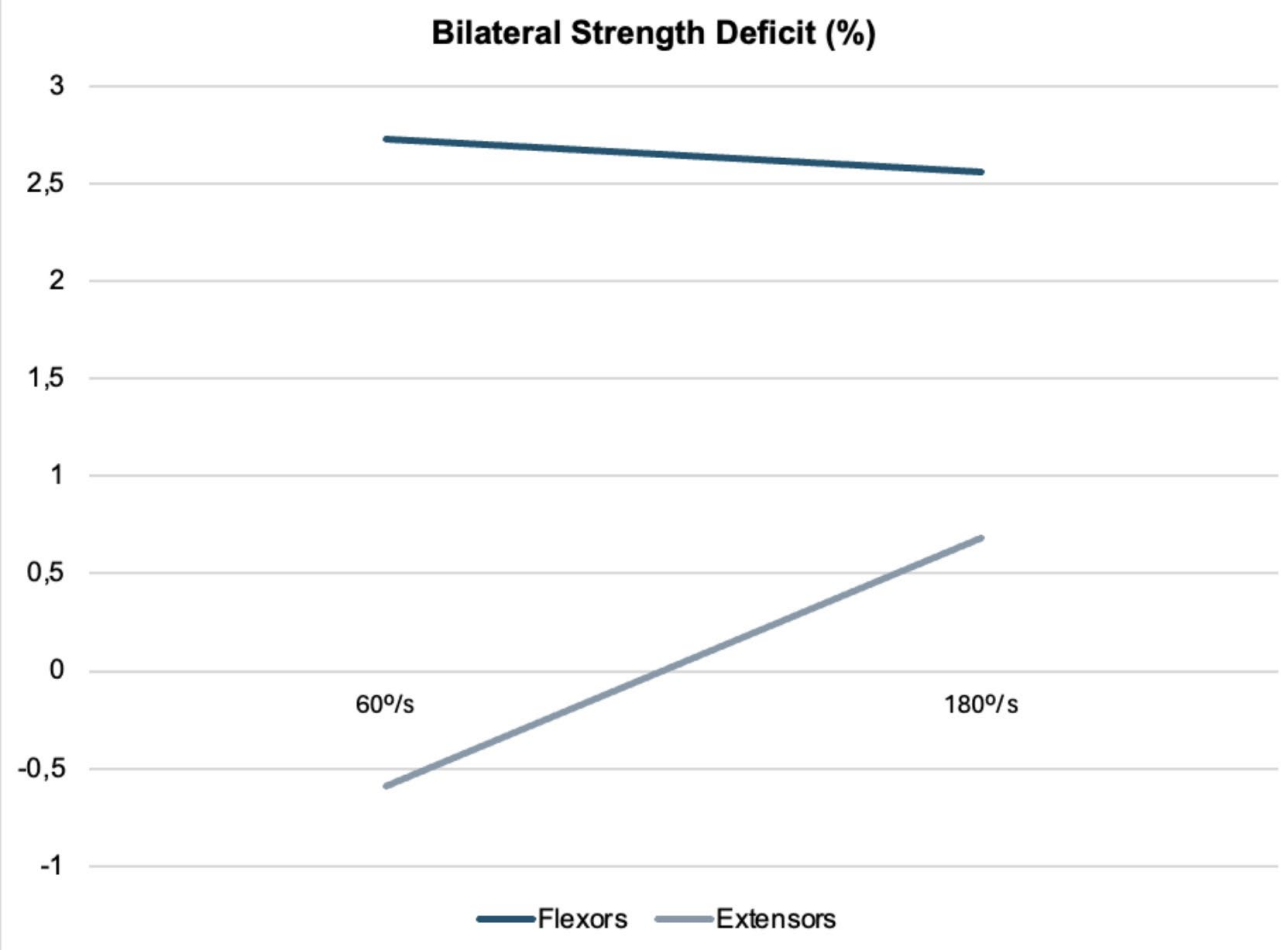
Bilateral strength deficit assessment in knee muscle strength.

Finally, Fig. 2 displays the results of the bilateral strength deficit analysis. Values ranged between 2.72 ± 12.66% and 2.56 ± 12.45% for KF and from − 0.59 ± 7.52 to 0.68 ± 8.81% for KE at 60º/s and 180º/s, respectively.

## Discussion

The first aim of this study was to examine knee muscle strength performance considering intra- and inter-limb asymmetries among female football players. The results indicate no significant intra-limb asymmetry between KF and KE performance. Regarding inter-limb asymmetry, although the mean values obtained in the PL performance were superior to the NPL, the differences were not statistically significant. Secondly, the present study investigated the relationships between knee muscle strength, body composition, and vertical jump performance. The results revealed a strong and positive correlation between KF and KE variables both at 60º/s and 180º/s and vertical jumps. In contrast, BF% presented a significant negative relationship with the CMJ and SJ performance.

In the football literature, particular attention has been given to evaluating the strength of KE and KF since it might have positive implications for both on-field performance and injury prevention^12^. In the injury topic, intra-limb asymmetry has been related to hamstring strains in male football players^23,24^. On the other hand, the evidence on strength imbalances and injury in female football is still limited. However, it should be underlined that muscle strength deficits are a potential predictor for lower extremity injury^6,13^, and an important variable to be included in players’ assessment and monitoring process^12^, which coaches and their staff should consider.

Among top-level female football players, no significant differences have been reported concerning intra-limb asymmetry at 60º/s isokinetic strength assessment^25,26^. When comparing the results of the current study with previous investigations, similar values for the H/Q ratio at 60º/s were observed (PL: H/Q ratio = 55.4 ± 3.6% and NPL: H/Q ratio = 55.4 ± 4.6% ^26^; PL: H/Q ratio = 58.7 ± 11.8% and NPL: H/Q ratio = 58.2 ± 6.4% ^25^). It should be noted that H/Q ratio values vary according to the angular velocity evaluated since the role of the KE in stabilizing the knee joint becomes more pronounced at higher velocities^27^. Thus, the higher H/Q ratio at the 180º/s condition would be expected and is consistent with previous research among top-level football players^25^. Notably, both in the current study and past research, the mean values of the H/Q ratio at 60º/s condition are lower than the 60% recommended in the literature, which might suggest an increased likelihood of hamstring injury^23^. On the other hand, the literature has emphasized that female football players are more susceptible to knee injury, particularly in the anterior cruciate ligament, compared to their male counterparts^28,29^. Besides the immediate consequences for players and clubs from the time lost, the cost of injury might affect players in the long term and compromise their future sports participation. Therefore, including monitoring strategies for knee muscle strength performance in the training process might play a substantial role in identifying the risk of injury and should be considered during the planning and periodization periods.

In addition to intra-limb asymmetries, bilateral strength deficits are also a concern for injury risk. According to the research, muscle strength differences within the normative data (less than 15%) may reflect the bilateral pattern of sport-specific activities^30^. However, values greater than 15% have been considered risk factors for injury and impaired biomechanics of athletic movements^31^. In the current study, scores of bilateral strength deficits did not reflect substantial inter-limb asymmetries, consistent with previous research on top-level female football players^25,26^.

According to the results, players body composition and vertical jump performance are within the ranges previously reported in the literature (48 to 72 kg for bodyweight, 155 to 174 cm for stature, 13 to 29% for BF%, and 28–50 cm for vertical jump tasks)^32^. When examining the relationships between body composition, isokinetic strength, and vertical jump performance, significant and negative correlations between BF% and vertical jump tasks were found. These results would be expected since the detrimental effect of BF% is apparent in tasks requiring projection (i.e., jumps) and rapid movements (i.e., change of direction, shuttle run, jumps)^20,21,33^. Since football game performance is characterized by its high-intensity actions, which are strongly supported by lower-body strength performance, avoiding the detrimental effects of BF% is of great importance. Based on this, multidisciplinary approaches within the training process organization, such as nutritional guidelines, should be recommended.

In contrast, KF and KE scores were significantly and positively correlated to vertical jumping, with the strongest correlations observed at the highest angular velocity, consistent with previous research in adolescent male football players^34^. Regardless of sex, the literature has described a significant influence of knee muscle strength in explosive strength ability^1^. Specifically, the present study findings indicate a significant relationship between KE and vertical jumps, as stated in male professional footballers^35^. To our knowledge, no data on these relationships was formerly found among female football players. Although both KF and KE are crucial for jumping performance, the literature has emphasized the role of KE during the take-off stage of vertical jumping^36^. The findings of the current study, combined with previous research data, underline strength training as an important part of footballers’ preparation.

This study presents some limitations that should be recognized. Firstly, a convenience sample of 27 participants was used. All players belonged to the same football club, and therefore, the findings should not be fully generalized to all female football players. Yet, importantly, the homogeneity of the sample regarding the club, training program, schedule, and organizational lifestyles, among others, allowed focusing on individual differences in the detailed measures obtained and their relationships. Secondly, this was a cross-sectional study, limiting the analysis of changes over time. However, with its appropriate feasibility, first explorations must start with cross-sectional data. Thirdly, it is worth noting that data regarding the menstrual cycle was not collected, which might have implications for the body composition and performance variables of female footballers^37^. Based on our initial insights, future follow-up studies can then be conducted to examine (i.e., seasonal variation in knee muscle strength, body composition, and vertical jump performance), allowing a complementary analysis and interpretation of the present results. Thirdly, the vertical jump is a multi-joint closed-motion kinematic chain task, and knee muscle strength assessed by an isokinetic dynamometer provides single-joint movement in an open kinematic chain^19^. Thus, measures of knee muscle strength might only provide partial information on the vertical jump performance. However, our measures have the advantage that they can be easily implemented and be important predictors of performance in male football^20,38^. Finally, details on players’ diets were not considered and should be included in future studies to provide a more in-depth analysis of body composition variables.

Even though, the current study results are of great interest to the female football training process, particularly considering the lack of data available in the literature on this topic. Since identifying intra- or inter-limb asymmetries has been reported as a potential for injury^7,8,13^, monitoring knee muscle strength might be a valuable tool for coaches and their staff in identifying possible injury risks. On the other hand, based on the football game’s high-intensity characteristics, the significant relationship between knee muscle strength and vertical jump highlights strength training as a crucial part of footballers’ preparation. Finally, the detrimental effect of BF% on explosive strength tasks indicates the need to consider multidisciplinary approaches, including nutritional guidelines, in the football training process.

Future studies to assess longitudinal data regarding body composition and lower-body strength performance are recommended to investigate seasonal variations in female football. Additionally, analyzing players’ physical fitness according to playing position would contribute to a more in-depth understanding of players’ profiles, which might sustain adequate training prescription.

## Methods

### Participants

A convenience sample of 27 semiprofessional female football players (age = 21.5 ± 4.9 years, bodyweight = 61.3 ± 7.8 kg, stature = 165.3 ± 5.7 cm) competing in the First Portuguese National Division (Liga BPI) were included in this study. Limb preference was the preferred leg to kick the ball^39^. Nineteen players had the right lower limb as the preferred one, while eight had the left limb as preferred. All players had more than five years of football training experience and were not injured for at least one month before data collection. Five players were previously submitted to surgery due to injury, and four related to the knee joint. The last surgery occurred two years before data collection, and players were fully recovered. Typically, players were involved in five weekly training sessions (mean duration of 90 min) and one match during the weekend. This study only included players fully participating in training sessions for at least one month before data collection.

### Body composition

Stature was measured to the nearest 0.01 cm using a stadiometer (SECA 213, Hamburg, Germany). Body composition was evaluated using a hand-to-foot bioelectrical impedance analysis (InBody 770, Cerritos, CA, USA) with participants in a fasted state during the early morning. Participants were barefoot and wearing only their underwear. On the platform, their feet were placed on the defined spots, and their arms were placed nearly 45º from their trunk until the assessment was concluded. Bodyweight, body fat percentage (BF%), and fat-free mass (FFM) were retained for analysis.

### Knee muscle strength

Knee muscle strength was measured using the Biodex System 4 Pro Dynamometer (Shirley, NY, USA). The isokinetic strength of knee extensors (KE) and knee flexors (KF) from the preferred leg (PL) and the non-preferred leg (NPL) was obtained at an angular velocity of 60º/s. Before data collection, participants engaged in a 5-min warm-up on a reclining bicycle (Technogym Xt Pro 600 Recline, Cesena, Italy) with intensity levels ranging from 4 to 5, maintaining a cadence between 50 and 60 rotations per minute. After the warm-up, participants were seated on the dynamometer following the manufacturer’s guidelines, adopting a standardized position of 85º hip flexion from the anatomical position. The dynamometer’s lever arm was aligned with the lateral epicondyle of the knee, and belts were used to stabilize the trunk, the evaluated thigh, and the leg. The range of motion was determined based on each participant’s maximum knee extension. Then, participants were asked to bend the knee until 90º of flexion. Calibration for gravity correction was individually performed at 30º of knee flexion^40^. Throughout testing, participants were asked to keep their arms crossed with the hand on the opposite shoulder holding the belts^41^. Verbal encouragement was provided, and three trials were allowed before testing started^42^. Subsequently, participants executed five repetitions of concentric contraction efforts of knee flexion and knee extension at 60º/s and 180º/s, with a 60-s interval after the sequence. The first round of assessments was performed with the preferred leg (PL) followed by the non-preferred leg (NPL) with a rest interval of 3-min. The analysis of isokinetic variables included the values of peak torque (PT), and peak torque normalized by bodyweight (PT/BW) for KE and KF for both legs. The H/Q conventional ratio was calculated by dividing the mean concentric KF PT by the mean KE concentric PT over the five repetitions^43^. Bilateral differences for KF and KE muscles were calculated using the following equation^26^:

$$Bilateral~strength~deficit = ~\frac{{PL~peak~torque - NPL~peak~torque}}{{PL~peak~torque}} \times 100\left( \% \right)$$

### Vertical jumps

The countermovement jump (CMJ) and the squat jump (SJ) were used to assess lower-body explosive strength. Both protocols included four data collection trials separated by 1-min rest. The assessment was performed using the Optojump Next (Microgate, Bolzano, Italy) system of analysis and measurement. In both tests, participants were encouraged to jump to maximum height. To ensure proper execution, each participant performed three experimental trials before data collection. Details regarding testing protocols can be found in previous literature^44^.

### Design and procedures

Data collection was conducted at the beginning of the season for two consecutive days in the morning (Fig. 1). All protocols were performed in a physical performance laboratory by experienced research team staff. Participation in this study was voluntary, and informed consent was obtained. The study’s procedures were approved by the Ethics Committee of the Faculty of Human Kinetics, University of Lisbon (approval code: CEIFMH N°34/2021) and followed the Declaration of Helsinki.

### Statistics

Descriptive statistics are presented as mean ± standard deviation. Preliminary analyses were conducted to guarantee the normality of the data using the Shapiro-Wilk test. The PL and NPL performance differences in knee muscle strength indicators were assessed using a paired-sample t-test. Finally, Pearson product-moment correlation was used to investigate the relationships between knee muscle strength and vertical jump performance. The strength of correlation was interpreted as follows: 0.1 < *r* < 0.3 (small), 0.3 < *r* < 0.5 (moderate), and 0.5 < *r* < 1.0 (large)^45^. All analyses were conducted using the IBM SPSS Statistics software 28.0 (SPSS Inc., Chicago, IL, USA). The significance level was set at 0.05.

## Data Availability

The data presented in this study are available upon request from the corresponding author.
